# Complete Genome Sequence of *Trueperella pyogenes*, an Important Opportunistic Pathogen of Livestock

**DOI:** 10.1128/genomeA.00400-14

**Published:** 2014-05-01

**Authors:** Vinicius S. Machado, Rodrigo C. Bicalho

**Affiliations:** Department of Population Medicine and Diagnostic Sciences, College of Veterinary Medicine, Cornell University, Ithaca, New York, USA

## Abstract

Here, we report the complete genome sequence of *Trueperella pyogenes* TP6375, a strain isolated from the uterus of a dairy cow affected with metritis. The complete circular genome is 2,338,390 bp and contains several genes needed for pathogenicity.

## GENOME ANNOUNCEMENT

*Trueperella pyogenes*, a Gram-positive, nonmotile, non-spore-forming, short, rod-shaped bacterium (1), is a common inhabitant of the urogenital, gastrointestinal, and upper respiratory tracts of many animal species (2–4). However, a physical or microbial insult to the host can lead to a variety of suppurative *T. pyogenes* infections, such as mastitis and uterine diseases in dairy cows (5, 6), liver abscesses in feedlot cattle (7) and high-producing dairy cows (8), pneumonia in pigs (9), and abscesses in various species of wildlife, such as musk deer (10). Infections caused by *T. pyogenes* are uncommon in humans and are usually linked to occupational exposure, because the organism is not part of the human normal flora (11). *T. pyogenes* can grow under aerobic or strictly anaerobic conditions, but it optimally grows in a CO_2_-enriched (7% CO_2_) atmosphere (1)

*T. pyogenes* is equipped with several known and putative virulence factors that are important for its pathogenic potential. Its primary virulence factor, pyolysin, is a potent cholesterol-dependent cytolysin and is associated with the tissue damage caused by *T. pyogenes* infection (1, 12). *T. pyogenes* also expresses a number of surface-exposed proteins, such as fimbriae, neuraminidases, and extracellular matrix-binding proteins, which are involved in adherence and mucosal colonization (1, 6, 13).

Here, we report the complete chromosome sequence of *T. pyogenes* strain TP6375. The strain was isolated from the uterus of a dairy cow affected with metritis and was subjected to whole-genome sequencing. The genomic DNA was extracted from an overnight culture using a PowerSoil DNA isolation kit, according to the manufacturer’s instructions. A library for sequencing was prepared with 2 µg of the extracted genomic DNA using a TruSeq DNA PCR-free LT sample preparation kit (Illumina), and paired-end sequencing was performed using the MiSeq reagent kit version 3 (600 cycles) with the Illumina MiSeq platform. The sequences were *de novo* assembled using the DNAStar SeqMan NGen (version 11.2.1.25) assembler. Genome annotation was done by the NCBI Prokaryotic Genome Annotation Pipeline.

The complete circular genome is 2,338,390 bp long, with a G+C content of 59.5% and 2,082 predicted genes; of those genes, 1,984 are coding sequences (CDS), 45 are pseudogenes, 1 is a clustered regularly interspaced short palindromic repeat (CRISPR) array, 6 are rRNAs, 46 are tRNAs, 1 is a noncoding RNA (ncRNA), and 15 are frameshifted genes. The genome encodes several known and putative virulence factors, including adhesion factors (1 collagen adhesion and 4 fimbrial proteins) and toxins (pyolysin, cytotoxin, and one other toxin). The complete genome sequence presented here will serve as a platform for identifying new genes that may contribute to pathogenicity, will advance our knowledge regarding the evolution, metabolism, and antibiotic resistance of this strain, and will serve as a template for future transcriptomic work.

### Nucleotide sequence accession number.

The annotated chromosome sequence of *T. pyogenes* strain TP6375 has been deposited in GenBank under the accession no. CP007519.
